# Status of inpatient pain therapy using the example of a general and abdominal surgery normal ward – a prospective questionnaire study to review a pain therapy algorithm (“real-world data”)

**DOI:** 10.1515/iss-2023-0016

**Published:** 2023-10-05

**Authors:** Michael Brinkers, Mandy Istel, Moritz Kretzschmar, Giselher Pfau, Frank Meyer

**Affiliations:** Division of Pain Therapy, Department of Anesthesiology and Intensive Care Medicine, University Hospital at Magdeburg, Magdeburg, Germany; Department of Cardiology, University Hospital at Magdeburg, Magdeburg, Germany; Department of General, Abdominal, Vascular and Transplant Surgery, University Hospital at Magdeburg, Magdeburg, Germany

**Keywords:** pain intensity, NSAID, opioids, psychotropic drugs, therapeutic algorithm

## Abstract

**Objectives:**

The mean pain intensity for inpatient consultations, for example in cancer patients, is known. However, the proportion of necessary consultations in the total volume of patients of a ward or a hospital, the general pain intensity in a surgical ward and the relationship between pain medication, length of stay and therapeutic success are unknown. The aim of the study was to examine surgical patients in a single normal ward subclassified into various groups (−/+ surgery, ICU stay, cancer, consultation for pain therapy etc.) during half a year with regard to their pain. For this purpose, the pain score (NAS) was recorded daily for each patient during the entire hospital stay and the change was assessed over the clinical course.

**Methods:**

In 2017, all consecutive new admissions to a normal ward of general surgery at a university hospital (“tertiary center”) were monitored over half a year according to a standardized procedure. Pain severity (measured by the “Numeric rating scale” [NRS] respectively “Visual analogue scale” [VAS]) from admission to discharge was recorded, as well as the length of stay and the administered medication. Patient groups were sub-classified as surgery, intensive care unit, cancer and pain consultation. An algorithm in two parts (part 1, antipyretics and piritramide; part 2, WHO-scheme and psychotropic drugs), which was defined years before between surgeons and pain therapists, was pursued and consequently used as a basis for the evaluation of the therapeutic success.

**Results:**

269 patients were included in the study. The mean pain intensity of all patients at admission was VAS 2.2. Most of the groups (non-cancer, intensive care unit [ICU], non-ICU, surgical intervention (=Operation [OP]), non-OP, pain intensity greater than VAS 3) were significantly reduced in pain at discharge. An exception in this context was patients with cancer-associated pain and, thus, initiated pain consultation.

**Conclusions:**

Since three quarters of the consultation patients also reported cancer pain, it might be possible that the lack of treatment success in both the consultation and cancer groups is associated with cancer in these patients. However, it can be shown that the successfully treated groups (without ICU-based course) had a mean length of stay from 4.2 ± 3.9 up to 8.4 ± 8.1 days (d), while the two unsuccessfully treated groups experienced a longer stay (mean_“cancer”_, 11.1 ± 9.4 d; mean_“consulation”_, 14.2 ± 10.3 d). Twenty-one consultation patients, in whom it had been intended to improve pain intensity, could not be successfully treated despite adapted therapy – this can be considered a consequence of the low number of patients. Since the consultation patients were the only patient group treated with part 2 of the algorithm, it can be concluded that part 1 of the algorithm is sufficient for a mean length of stay up to 9 days. For all patients above this time point, a pain consultation with adaption of medical treatment should be considered.

## Introduction

In surgery, pre-, intra- and post-operative pain management forms the basis for greater patient satisfaction and can be helpful in avoiding pain-associated sequelae and complications [1]. Nevertheless, problems and deficits were repeatedly described in the past with regard to pain therapy despite established therapy procedures. Postoperative pain was often judged to be undersupplied [2, 3]. According to Schiek et al. [4], direct pain assessment by physicians during ward rounds takes place in only 20 % of cases, and nursing staff inquire about pain in only 16 % of patient consultations.

In a US study from 2011, less than 50 % of patients with postoperative pain received adequate pain therapy [5]. Therefore, authors have repeatedly noted a considerable need for optimization at many hospitals up to recent times [5].

In 2017, the pain outpatient clinic of the University of Magdeburg investigated the quality of analgesic administration in consults using the example of cancer patients. Weaknesses were identified in 375 patients.

Hardly any slow-release opioids were given prior to the pain consultation. The non-retarded opioids were given only when needed and only subcutaneously or intravenously. Co-analgesics were hardly used [6].

In the pain outpatient clinic at the University Hospital of Magdeburg (Germany), a therapeutic algorithm has been developed in interdisciplinary cooperation with surgeons and inaugurated to clinical practice to optimize pain therapy [7]. The algorithm consists of two parts: the first consists of three days of NSAIDs plus unretarded opioids, then, as part two, a switch to retarded opioids plus co-analgesics.

In a recent publication, Stramer et al. found that after major surgery and prolonged pain, NSAIDs may be administered safely for longer than three days [8].

Additionally, more than 300 pain-associated consultations in cancer patients over 3 years were evaluated in a retrospective study. Therein, despite relatively high pain scores (mean VAS 5.5), successful pain therapy – by using the second part of the algorithm – was achieved in 70 % (=clinical success rate) of cases [8].

The substantial disadvantage of that approach and, in addition, of many other studies was that only consultations were considered, documented and evaluated. No data exist for the proportion of pain consults on a normal ward of general surgery. In addition, there is no reliable value known for the general pain level on a general surgery ward. Furthermore, no data was available on how many patients the described algorithm was applied to.

Therefore, the following questions were raised:

1. What is the level of pain on a typical general surgery ward?
2. Was it possible to achieve sufficient pain reduction in patients from admission until discharge?
3. Were there any special features with regard to the success of pain therapy in certain sub-classified groups of patients?
4. Pain consultations:
  (a) Do the pain consultations reflect the clinical reality of pain reported on a surgical ward?
  (b) How can patients who were brought to a pain-associated consultation be characterized?
5. Are the recommendations of the pain therapy algorithm of the university of Magdeburg realistic, and how long can the first part of the algorithm (NSAID plus unretarded opioids) be applied?

**Hypothesis:**

Standardized continuous observation of all patients in a university general surgery ward over a longer period of time provides clarity about the level of pain in the ward, the proportion of pain consults required and the medications needed for successful pain management. Most patients have a pain intensity of VAS≥4. Nevertheless, successful pain therapy using retarded opioids is possible in most patients. In about 10 % of cases, pain consultations become necessary.

## Materials and methods

### Study design

The Study was approved by the ethics committee of the University Hospital Magdeburg.

A prospective questionnaire study was conducted on ward 1 of the Department of General, Abdominal, Vascular and Transplant Surgery of the University Hospital at Magdeburg (Germany) during the time period from 01/01/2017 to 06/30/2017.

All medical and nursing staff of the ward were informed about the data collection before the start of the study period.

At the beginning of a hospital stay, the nursing staff handed out a standardized questionnaire (see Appendix 1) for the assessment of the individual pain condition to each patient on the day of admission.

During the entire inpatient stay, the nursing staff recorded the patients’ pain intensity twice a day using the “visual analogue scale” (VAS) and recorded the values in the ward’s documentation system. This pain recording was conducted in conjunction with the daily blood pressure, pulse and body temperature checks.

If a patient had to be transferred to the intensive care unit (ICU) of the university hospital during the hospital stay, the daily VAS values were not recorded during these ICU days. The recording of pain scores was continued when the patient was transferred back to the normal ward. If a patient was transferred to another ward of the hospital, this was regarded as a discharge, the data recording was terminated and it was regarded as a new admission in the event of a possible transfer back.

### Data collection

The pain intensity (VAS) was taken subsequently from the inpatient documentation system.

The starting data relevant to pain therapy were taken from the questionnaire handed out by the nursing staff at the beginning of the hospital stay. This included pain localization, pain intensity, onset, course, rhythm and any factors influencing the pain, concomitant symptoms, pain medication and pain quality.

The pain medication at the beginning and in the course of the clinical stay was also taken from this documentation system. Pain medication was recorded under assignment to the subgroups antipyretics (NSAIDs), weak opioids, strong opioids and psychotropic drugs.

Other data relevant to this study were found in the patient records and the patients’ medical letters. These included the diagnosis, whether a surgical intervention had taken place, the day of surgery and the length of stay. Length of stay was subdivided into preoperative, postoperative, total and ICU days spent at the university hospital.

The diagnoses treated were assigned to different headings, which in turn were based on specific body regions. The generic terms are:

– Head/neck, – Gastrointestinal tract,
– Hepatobiliary system, – Other abdominal organs,
– Lymphatic system, – Skin/bone/connective tissue and
– Other(s).

Furthermore, the date of discharge, any malignancy present (equated with the term “cancer diagnosis” in this study) were extracted from the patient records and letters.

For data collection, the documentation system of the outpatient clinic (Pain Division) was also used and provided information about consultations and medication changes by the pain therapists.

### Inclusion and exclusion criteria

January 2017 was used as trial month. Patients who had been admitted to the ward during the period from 01/01/2017 to 01/31/2017 were, therefore, not subsequently included in the scientific evaluation.

Furthermore, the scientific evaluation did not include individuals for whom the diagnosis was still unknown and who had been admitted to the inpatient unit exclusively for a non-interventional, non-surgical diagnostic procedure.

Patients from other wards or medical disciplines, who were temporarily housed on surgical ward 1 due to a shortage of beds, were also not included in the analysis.

Patients undergoing major abdominal surgery at the University Hospital of Magdeburg usually receive an epidural catheter with i.v. or s.c. piritramide (Dipidolor^®^) for breakthrough pain prior to surgery and for a maximum of the first three post-operative days. The epidural catheter was not considered in the evaluation.

Furthermore, the exclusion criteria were the non-existence of the informed consent and the refusal to participate in the study.

### Ethics

Data collection did not exceed the usual level of an inpatient admission. However, since the patient data were obtained and used prospectively for scientific evaluation, the project was presented to the institutional ethics committee (Ref: 180/16).

As a result, patients were provided with an information sheet and informed consent form at the beginning of their inpatient stay, in addition to the pain management admission form. This information sheet (see Appendix 2, Part 1) included – pseudonymized – the general procedure for data collection on the ward, the requirements for the patient, the indication that no personal advantages or disadvantages would result from participation or non-participation, the information that the data would be recorded in a register.

With the informed consent form (see Appendix 2, Part 2), the patient confirmed by signature that she/he had received, read and understood the information sheet.

They consented to the pseudonymized data collection and were additionally informed that revocation of consent is possible at any time without giving reasons.

### Questionnaire

The questionnaire used (see Appendix 1) was given to each patient on admission to hospital.

The parameters collected in the questionnaire were:

– Pain localization
– Pain intensity
– Pain quality
– Previous course of pain
– Concomitant symptoms
– Current pain medication with dosage and time of intake.

### Assessment of pain intensity according to the “Visual Analogue Scale” (VAS)

The visual analogue scale (VAS), respectively, numeric rating scale (NRS), in the field of pain therapy is actually a range of semiquantitative values extending from zero to ten. VAS 0 was defined as “no pain” and VAS 10 as “pain intensity at which the patient would jump out of the window”.

In addition, for comparability with international literature, pain was classified as mild (VAS 1–3), moderate (VAS 4–6) and severe (VAS 7–10).

### Statistics

The software IBM^®^ SPSS Statistics, version 24 (SPSS^®^, IBM Corporation, Armonk, New York, USA) was used for statistical data collection and analysis.

First, a descriptive description of the study variables was performed. Special interest was given to diagnosis, pain quality, pain medication and pain intensity. Results were reported as frequencies in absolute and percentage terms and as position and ratio parameters (mean, standard deviation).

The Wilcoxon paired difference test was used to compare pain levels at admission and discharge (overall and separately for different subgroups). Comparisons of pain severity and other quantitative data regarding different subgroups were made using the Mann–Whitney U test. Contingency table analyses were performed to show associations between qualitative variables, using Pearson’s chi-square test.

A probability of error of α=0.05 was assumed as the significance level for the statistical tests.

### Statement

The study was conducted in accordance with the guidelines of the 1964 Declaration of Helsinki for Biomedical Research of the “World Medical Association” and its further implementing regulations as well as on the basis of “Good Clinical Practice” and “Good Research Practice”.

## Results

### General

**Figure 1:**
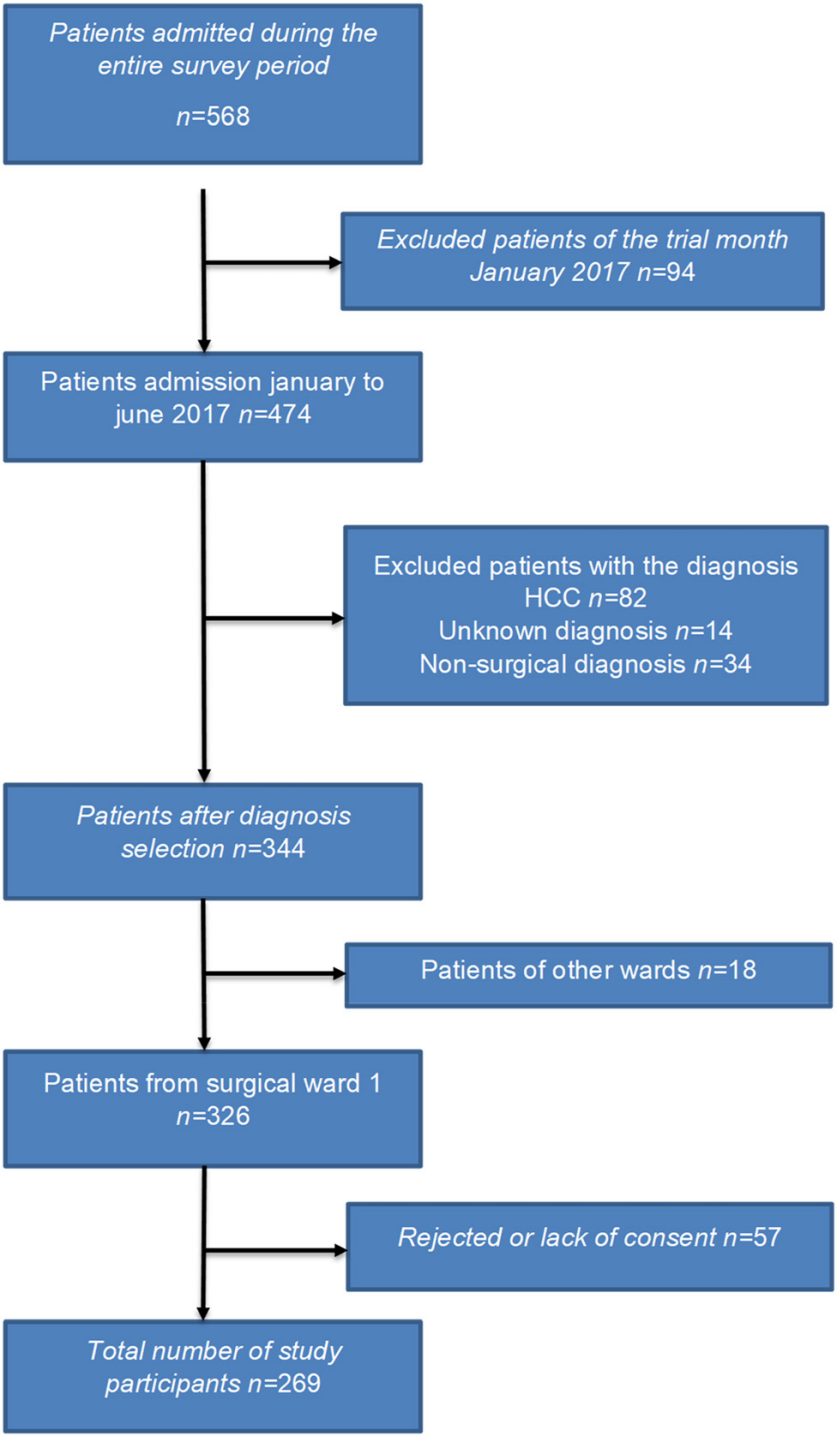
Flow chart of patients primarily enrolled and finally analyzed.

Of the original 568 patients enrolled during the study period, only 269 could be included in the study at the end for final and reliable analysis (see Figure 1).

The general patient data are presented in Table 1.

**Table 1:** Demographic data of the study participants.

	Total	Female	Male
Number of patients: MV, %	269 (100.0)	140 (52.0)	129 (48.0)
Age, y: MV^a^ [±SD]^b^	55.4 [±16.8]	54.4 [±17.9]	56.4 [±15.4]
Length of stay (days): MV [±SD]	7.2 [±7.4]	7.0 [±6.6]	7.4 [±8.2]
*Surgical procedures*			
Yes: n^c^, %	193 (71.7)	100 (71.4)	93 (72.1)
No: n, %	76 (28.3)	40 (28.6)	36 (27.9)
*Actual cancer diagnosis*			
Yes: n, %	95 (35.3)	53 (37.9)	42 (32.6)
No: n, %	174 (64.7)	87 (62.1)	87 (67.4)
*Localisation of diagnosis*			
Head/neck: n, %	19 (7.1)	12 (8.6)	7 (5.4)
Gastrointestinal tract: n, %	61 (22.7)	33 (23.6)	28 (21.7)
Hepatobiliary system: n, %	67 (24.9)	33 (23.6)	34 (26.4)
Other visceral organs: n, %	37 (13.8)	21 (15.0)	16 (12.4)
Lymphatic system: n, %	11 (4.1)	5 (3.6)	6 (4.7)
Skin, bones, connective tissue: n, %	42 (15.6)	14 (10.0)	28 (21.7)
Others n, %	32 (11.9)	22 (15.7)	10 (7.8)

^a^MV, mean value; ^b^SD, standard deviation; ^c^n, numbers.

The subgroups, the pain intensity and the length of stay are presented in Table 2.

**Table 2:** Overview of the individual study groups as well as the patients in total.

Group	n^a^	VAS^b^	MV^c^ (±SD^d^)	Statistics	Length of stay in days (±SD^d^)
Total	269	Ad admission	Ad admission 2.2 (±2.8)	p<0.001	7.2 (±7.4)
		VAS 0: n=138			
		VAS 1–3: n=60			
		VAS 4–6: n=41			
		VAS 7–10: n=30			
		Ad discharge	Ad discharge 1.2 (±1.7)		
		VAS 0: n=153			
		VAS 1–3: n=87			
		VAS 4–6: n=27			
		VAS 7–10: n=2			
Intensive care unit (ICU)	Without ICU N=214		Without ICU^e^	p<0.001	5.2 (±5.7)
			A: 2.3 (±2.9)		
			D: 0.9 (±1.6)		
	With ICU N=55		With ICU^e^	p=0.030	14.8 (±8.1)
			A: 2.0 (±3.0)		
			D: 1.3 (±1.7)		
Cancer	Without Cancer N=174		Without Cancer	p<0.001	5.0 (±4.9)
			A: 2.6 (±2.9)		
			D: 1.2 (±1.6)		
	With Cancer N=95		With Cancer	p=0.442	11.1 (±9.4)
			A: 1.3 (±2.3)		
			D: 1.1 (±1.6)		
Operation (OP)	Without OP N=76		Without OP	p<0.001	4.2 (±3.9)
			A: 3.0 (±2.9)		
			D: 1.2 (±1.8)		
	With OP N=193	Post OP: VAS 0: n=32 VAS 1–3: n=62 VAS 4–6: n=56 VAS 7–10: n=10	With OP	p=0.004	8.4 (±8.1)
			A: 1.9 (±2.8)		
			D: 1.2 (±1.6)		
VAS≥4	N=71	VAS 4–6: n=41	A: 6.3 (±1.9)	p<0.001	5.9 (±5.4)
		VAS 7–10: n=30	D: 1.8 (±1.9)		
Consults	N=21		A: 2.6 (±3.7)	p=0.238	14.2 (±10.3)
			D: 1.6 (±2.1)		

^a^n, numbers; ^b^VAS, visual analoge scale ≙ NRS (Numeric rating scale); ^c^MV, mean value; ^d^SD, standard deviation; ^e^A, admission; D, discharge.

One third of the patients did not undergo surgery.

Most patients experienced no ICU stay despite surgery. Out of the 193 operated patients, only 51 (26.4 %) were admitted to the ICU.

#### Level of pain

Most subgroups studied had mean pain intensity below VAS 3.

Non-cancer patients had significantly higher pain intensity scores on admission than cancer patients (p<0.001).

Of the 193 operated patients, pain levels were assessed in 160 patients on the day after surgery. Among these, 32 reported no pain. Post-operatively, the majority of patients had mild (n=61, 38 %) and moderate (n=56, 35 %) pain. Only 10 patients (6.4 %) had severe pain during post-operative course.

Pain level on admission is higher in non-cancer patients than in cancer patients (2.6 + 2.9 vs. 1.3 + 2.3).

#### Sufficient pain reduction

##### Mild pain

A significant pain reduction was achieved in patients with mild pain in the following groups: Total group, patients with and without ICU stay, patients without cancer, patients with and without surgery.

The mean pain intensity from admission to discharge was not reduced in 21 pain consults, (2.6 ± 3.7 vs. 1.6 ± 2.1; *p*=0.238), and in the 95 cancer patients (1.3 ± 2.3 vs. 1.1 ± 1.6).

##### Moderate pain

Pain reduction was achieved in all patients (n=41) with moderate pain (4.9 ± 0.8 vs. 1.8 ± 1.9).

##### Severe pain

There were 30 patients with severe pain at the time point of admission. The pain reduction at discharge was reduced (8.2 ± 1.1 vs. 1.7±0.8; p<0.001).

#### Special features with regard to the succesfull therapy

Patients successfully treated (outside ICU) had shorter lengths of stay than those unsuccessfully treated:

Length of stay in patients without operation was 4.2 ± 3.9 and in the operated patients 8.4 ± 8.1 days.

In comparison, the length of stay of the two unsuccessfully treated groups was:

In cancer patients 11.1 ± 9.4 and in non-cancer patients: 5.0 ± 4.9 days (*p*>0.001) (cancer patients vs. Operated patients: p=0.01).
In the 21 pain consults 14.2 ± 10.3 days (consultation patients vs. operated patients: p<0.005).

The exception to this is the successfully treated ICU patients with a length of stay of 14.8 ± 8.1 days.

#### Pain consultations

##### Pain consult patients (Tables 2 and 3)

At the beginning of the study period, the mean pain intensity of the consultation patients was 2.6 and could not be reduced up until discharge.

**Table 3:** Characteristics of consultation vs. all patients and patients with VAS>4.

Parameter	Consultation patients, n=21 MV (±SD)^a^	All patients, n=269 MV (±SD)	Patients with VAS≥4, n=71 MV (±SD)
Length of stay, days	14.2 (±10.3)	7.2 (±7.4)	5.86 (±5.35)
Proportion of operations, %	85.7	71.7	57.7
Proportion of cancer, %	76.2	35.3	21.1

MV, mean value (±standard deviation).

Patients who received a pain consult form a special group. They had a significantly higher proportion of *cancer patients* and a significantly longer hospital stay than *the overall group*, while patients with *VAS≥4* had the lowest proportion of cancer patients and the shortest length of stay of these three groups (patients VAS≥4 vs. consultation patients: p<0.001).

#### Pain therapy algorithm

All patients outside the consultation group did not receive sustained-release opioids but received unretained piritramide.

239/269 Patients (88.85 %) received a WHO level 1 medication after admission; 200/269 (74.35 %) received piritramide (unretarded piritramide i.v.) or opioid s.c.

– Specifically:

Patients without surgery received antipyretics in 71.1 % and a strong opioid (piritramide) in 39.5 % during the stay.

More than two-thirds of patients received an unretarded strong opioid in the following groups:

A total of 95.9 % of patients with surgery received antipyretics and 88.1 % piritramide during the clinical course whereas 93.0 % of patients with VAS≥4 received antipyretics and 73.0 % piritramide.

The patients with ICU stay received antipyretic in 93 % and a strong opioid in 84 % of cases.

The fundamentally different medication of the therapy algorithm (Appendix 3, part2) was followed only within the consultation group.

The consultation patients received sustained-release opioids (morphine sulfate) in 95.2 % – and (despite discontinuation in the consultation) continued to receive antipyretics in 95.2 %. Only 18 patients received psychotropic drugs. These were prescribed in context of the pain therapy consultations.

## Discussion

“Not only does inadequate pain management following surgery result in increased morbidity and mortality, but it also may delay recovery, result in unanticipated readmissions, decrease patient satisfaction and lead to chronic persistent postsurgical pain [9].” The mean pain level of the total group at admission was VAS 2.2 ± 2.8. Pain could be reduced in the total patient group, patients with and without ICU stay, patients without cancer and patients with and without surgery. Taking into account the questions posed at the beginning, the results can be discussed as follows.

### Presence of algorithm

As late as 1998, Neugebauer et al. [10] found that only 19.1 % of 1,000 hospitals/surgical departments in Germany had a written therapeutic regime, only 11 % performed postoperative pain measurements with VAS and 33 % of the surgeons only considered pain therapy when the patient complained. Neugebauer et al. [10] concluded that pain management of many surgical clinics in Germany was ineffective, inadequate and lacked the necessary organizational and scientific background.

At the University Hospital of Magdeburg, this issue was specifically addressed by first developing an algorithm for anesthesiologists. In 2008, the first manual on pain therapy suitable for a lab coat pocket was published. It was intended to help the pain therapy consultant to treat pain according to a certain basic pattern. This was followed by the creation of an extended algorithm mentioned above, which also defined the actions to be taken by the primary treating surgeons during the first days after surgery but still before a possible consultation [7].

### Pain measurement

Previous work on postoperative pain has varied widely in design. In 2004, Strohbuecker et al. [7] studied pain intensity in a German university hospital (excluding ICU, psychiatry, gynecology and pediatrics) in 561 patients (with operative and conservative [non-operative] treatment); 50 % of them reported pain during the interview.

In a multicenter study of 3,251 patients, Maier et al. [11] found that 80 % of all hospitalized patients had pain. Of these, 2,252 were surgically treated patients. The patients were interviewed only on the first post-operative day. Of these, 12.4 % reported no (resting) pain, 21.3 % reported moderate pain and 8.2 % reported severe pain. Thereby, the middle value of resting pain across all postoperative patients was 2.6 (±2.4). Fletcher et al. [1] also studied 3,120 surgically treated patients from 21 European hospitals with regard to pain – also measured only on postoperative day 1. A second pain survey took place 6 and 12 months later via e-mail or telephone interview. In the study by Bialas et al. [12], only maximum and minimum pain was measured.

### Pain classification

The aim of the present study was to examine the population of patients in a single surgical ward during half a year with regard to their pain. For this purpose, the pain score (VAS) was recorded daily for each patient during the entire inpatient stay.

It is first important to note that 51.3 % of patients in the studied ward reported no pain on admission.

According to the “German Pain Society”, patient-reported VAS≥4 is defined as pain and >6 as severe pain [13]; VAS <4 is classified as non-relevant. Pain was defined by Maier et al. [11] and Sabatowski et al. [14] in the same way as done here in the presented study. Cleeland et al. [15] also defined mild pain as VAS 1–3, but like Breivik et al. [16], severe pain was only defined as VAS 8 or higher.

– **Low pain levels**

Almost all groups studied (not just the total) reported mild pain during hospital stay (mean_VAS_ 2.2). This was true not only for the ICU patients but also for the cancer patients. This corresponds to the values found by Maier et al. [11] in 2,252 surgically treated patients (2.6 ± 2.4).

The proportion of cancer patients in the consultation patient group of the present study was n=16/21 (76.2 %), more than twice as high as in the total group. However, in our own study [6] of 375 cancer patients with consultations for pain therapy, the pain intensity score was 5.0 (±2.39), which was also much higher than the score of Maier et al. [11] (namely, 2.0). It is not possible to explain why the values were now so low overall and particularly in case of cancer patients.

– **Distribution of pain severities**

Breivik et al. [16] studied 4,839 patients with chronic pain from 15 European countries by telephone interview. 66 % had moderate, 34 % severe pain. In Strohbuecker et al. [17], 58 % reported moderate and 36 % severe pain at a university hospital. In the present study, 73.6 % had no or mild pain, 15.3 % had moderate pain and 11.2 % had severe pain on admission.

– **Postoperative pain**

Apfelbaum et al. [2] studied 250 randomly selected surgical patients in the United States; 82 % reported pain post-operatively. Of these, 13 % reported mild pain, 47 % moderate pain and 39 % severe pain during the first 2 weeks after surgery. In the study by Gramke et al. [18], 26 % of patients reported moderate to severe pain (VAS>4) on the day of surgery. On day 1 after surgery, only 21 % still had moderate or severe pain. In the study by Sommer et al. [19], 41 % of study subjects reported pain >VAS 4.

In the present study, the majority of patients had mild (38 %) and moderate (35 %) pain post-operatively. Only 6.4 % had severe post-operative pain. Thus, 40 % of patients had moderate to severe pain post-operatively. This is slightly higher than the German study from 2010 but lower than the Austrian values from 2017 [20]. In German work [11], up to 30 % have moderate to severe pain. In Austrian work, 20–40 % have severe pain after surgical procedures [20].

### Pain therapy by surgeons

In the algorithm of the University Hospital of Magdeburg, there are two stages: Therapy of the first three days and therapy during the following days (Appendix 3). During the first three days, individuals were treated with anti-inflammatory drugs and piritramid. The treatment of the following days should follow the algorithm part 2 with slow-release opioids and psychotropic drugs if the pain persists.

In a US study from 2011 [5], 80 % suffered from postoperative pain, but less than 50 % received adequate pain therapy. Similar deficits are deplored in German studies [3]. In the study by Lorentzen et al. [21], 50 % of patients complaining of severe pain received strong opioids, 73.3 % received weak opioids and 100 % received antipyretics.

A total of 239 of 269 (88.8 %) patients in the present study received a WHO level 1 drug after admission, and 200/269 (74.3 %) received (unretarded) strong opioids.

However, the medications given were within the algorithm already published elsewhere [7]: piritramide as an unretarded opioid and antipyretics (ibuprofen, metamizol) during the first postoperative days. Thus, under unretarded opioids and NSAIDs (part 1 of the algorithm), sufficient pain reduction was achieved in the majority of patients during the first five days, with a maximum 9 days. This was also the case in the subgroups of patients without ICU stay, patients without cancer lesions as well as patients with and without surgery.

Cancer patients did not receive sufficient pain therapy due to a lack of prescribed sustained-release opioid (in the context of a consultation). A total of 95 patients with a tumor lesion were treated on the surgical ward. Therefor, 80 cancer patients did not get a pain consult at all to optimize pain treatment. The question of why could not be satisfyingly answered.

Only 7.8 % of patients were prescribed sustained-release opioids – these were the 21 pain consults.

Only 18 patients (6.7 %) received psychotropic drugs according to part two of the algorithm in the further course. These patients were also exclusively from the consultation group.

In its entirety, the pain therapy on the investigated ward corresponds to the algorithm agreed upon between pain therapists and surgeons [7].

### Consultations: pain management by the pain therapists/anesthesiologists

Only in 21/289 patients (7.8 %), a pain-consultation was requested by the ward. Thus, the consultation rate for pain patients is higher than other consultation rates in the CL (consultation-liaison) literature from psychiatry and palliative care. It is known from psychiatric literature that while 40 % of patients in German hospitals have a mental disorder, only 2.5 % receive a consult [22]. It is known from work on both palliative medicine and psychiatry that this can be increased to 5 % if primary treating physicians know the person of the consultation service [23]. Nevertheless, the percentage of consultations could have been much higher. Why this did not happen remains unclear at presence. However, the high proportion of cancer patients in the consultations (n=16/21) shows that there is a potential for further consultations. If the remaining 79 cancer patients, who also were not been treated successfully, would have also received a pain consult, the entire group might have been successfully treated as shown in the previous study [7]. Overall, 100 patients (the 21 consultations and the remaining 79 cancer patients) and thus nearly 40 % (37.2 %) should have received a pain consult. Probably only the “difficult” long-stayers among the cancer patients received a pain consult.

In addition, it is interesting to note that, despite discontinuation of antipyretics by the consultant, subsequent data processing revealed that these were nevertheless continued to be given as requested by the nursing staff.

### Therapeutic success according to algorithm

According to Table 3, the impression can be obtained that the proportion of cancer patients determines the success of therapy. This is because the proportion of cancer patients in consultations is more than twice as high as in the total group and the group of patients with VAS≥4 (Table 3). However, our own previous study [6] of pain consultations in more than 300 cancer patients has shown that cancer patients can be successfully treated.

Surgical patients with a mean hospital stay of up to 8 days could successfully be treated for pain with part one of the algorithm (NSAIDs with piritramide).

In a recently published recommendation by the German Pain Society, the German Society of Anesthesiology and Intensive Care Medicine and the German Society of Surgery, the authors suggested that NSAIDs may be taken for longer than three days postoperatively for persistent pain [8].

Although antipyretics were discontinued by the pain therapist in the consults (they are not part of the algorithm, Appendix 3, part 2), they were nevertheless continued by the ward in this study. This circumstance therefore also suggests that collaboration between ward staff and pain service needs to be improved.

Overall, the therapy algorithm with its two parts is realistic. What was surprising, however, was that the first part of the algorithm occupies such a large space while allowing successful pain management.

The hypothesis was only partially confirmed.

Most of the patients in the general surgery ward received successful pain therapy. However, it could not be clarified why the pain level on admission is so low (especially in cancer patients lower than in non-cancer patients). It could not be clarified why retarded opioids were not used outside the consults.

### Limitations

The ICU stay was not part of this investigation. The patients with stay on ICU were not evaluated as equivalent to the other subgroups because they were not continuously under the observed pain therapy on the normal ward. Therefore, it cannot be clarified how the ICU patients could be successfully reduced in pain despite a longer duration of stay of >9 days.

It was not recorded how long piritramide and antipyretics (part 1 of the algorithm) were given during the stay on the normal ward. However, based on the experience obtained in the evaluation of cancer consults [6], it must be assumed that outside the 21 consults considered, the remaining patients received the combination of NSAIDs plus non-retarded opioids until discharge.

The reasons for a pain therapy consultation could not be deduced. Possibly, the consultation request had an association with the length of hospital stay of the patients (mean hospital stay, 14 days). There is no algorithm for pain consults to date.

Despite cooperation with the nursing staff, the pain intensity was inquired postoperatively only in 169 of the 193 operated patients (87.6 %). In this context, a study by McDonald et al. is interesting [24]:

“The majority described avoiding or delaying communicating their pain at some point during their hospitalization. Reasons for decreased pain communication included:

– Not wanting to complain,
– Not wanting to take the provider away from other patients,
– Avoiding unpleasant analgesic side effects (and)
– Not wanting to take drugs.

Postoperative patients may be unclear about their role in pain management.”

## Conclusions

With the agreed algorithm, pain reduction was achievable in 174 non-cancer patients (64.7 % of the 269 patients studied). This was true for mild as well as moderate to severe pain. Sustained-release opioids and psychotropic drugs were not necessary for therapeutic success at short duration.

A pain consult should be considered if the patient is treated for pain for more than 9 days as a change in medication to sustained-release opioids and psychotropic drugs must be considered.

Increased attention should be paid to establishing a pain consult in cancer patients. The administration of antipyretics or medication on demand has not yet been clearly established.

For the first part of the therapy algorithm, adding a tolerance limit up to a 9 days post operatively to the duration of therapy with NSAIDs and nonretarded opioid seems feasible.

Why patients with an ICU stay were still successfully treatable despite a long length of stay requires further research.

## Supplementary Material

Supplementary Material
