# Supplementary material for: Status of inpatient pain therapy using the example of a general and abdominal surgery normal ward – a prospective questionnaire study to review a pain therapy algorithm (“real-world data”)
Source: Innov Surg Sci. 2023 Oct 5;8(2):73–82. doi: 10.1515/iss-2023-0016 (PMC10696937; doi:10.1515/iss-2023-0016)
Supplement: Supplementary file 1 — Supplementary Material [file j_iss-2023-0016_suppl_001.docx]

**Appendix 1** - Pain value registration sheet documenting medical history on pain


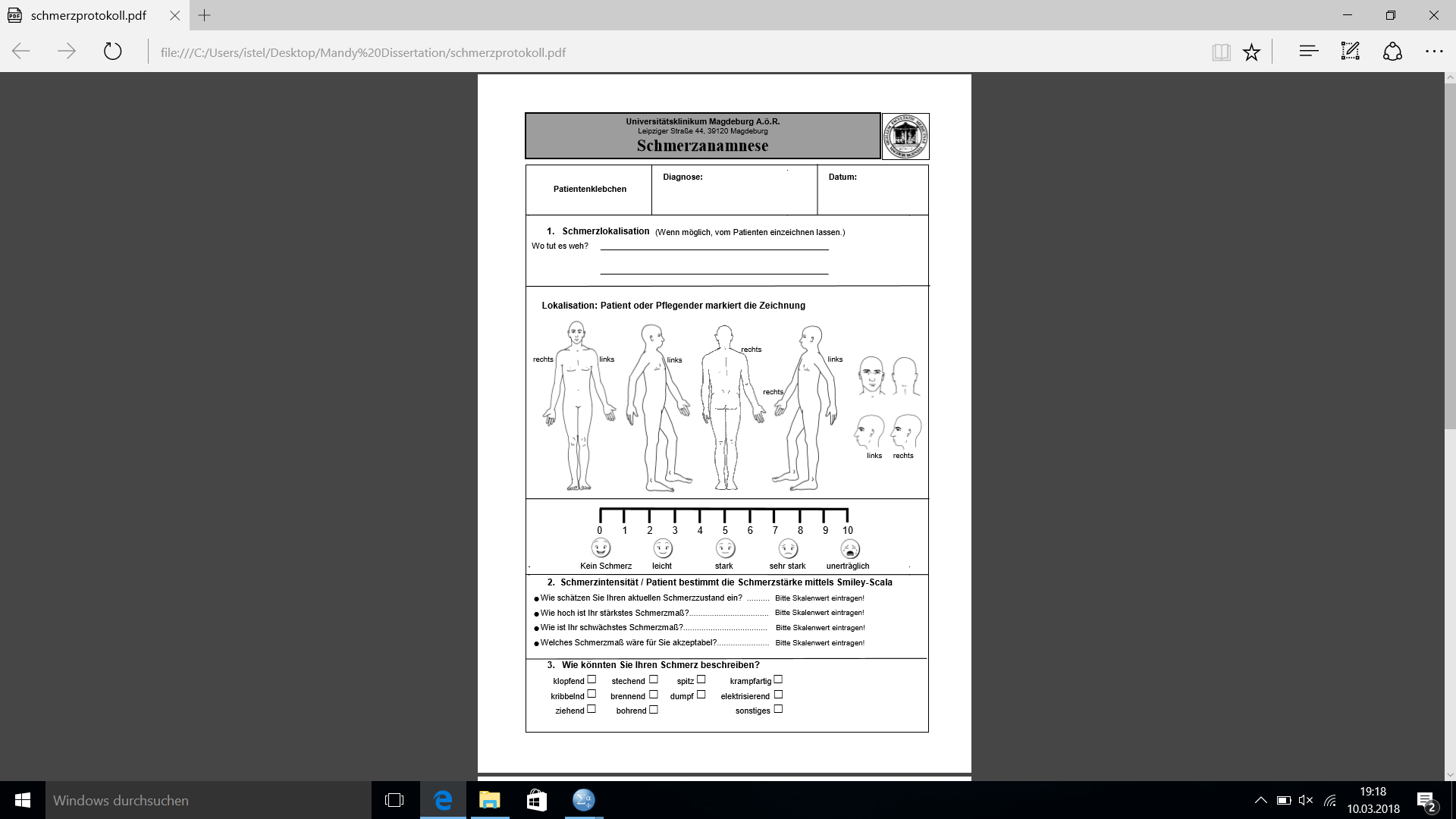


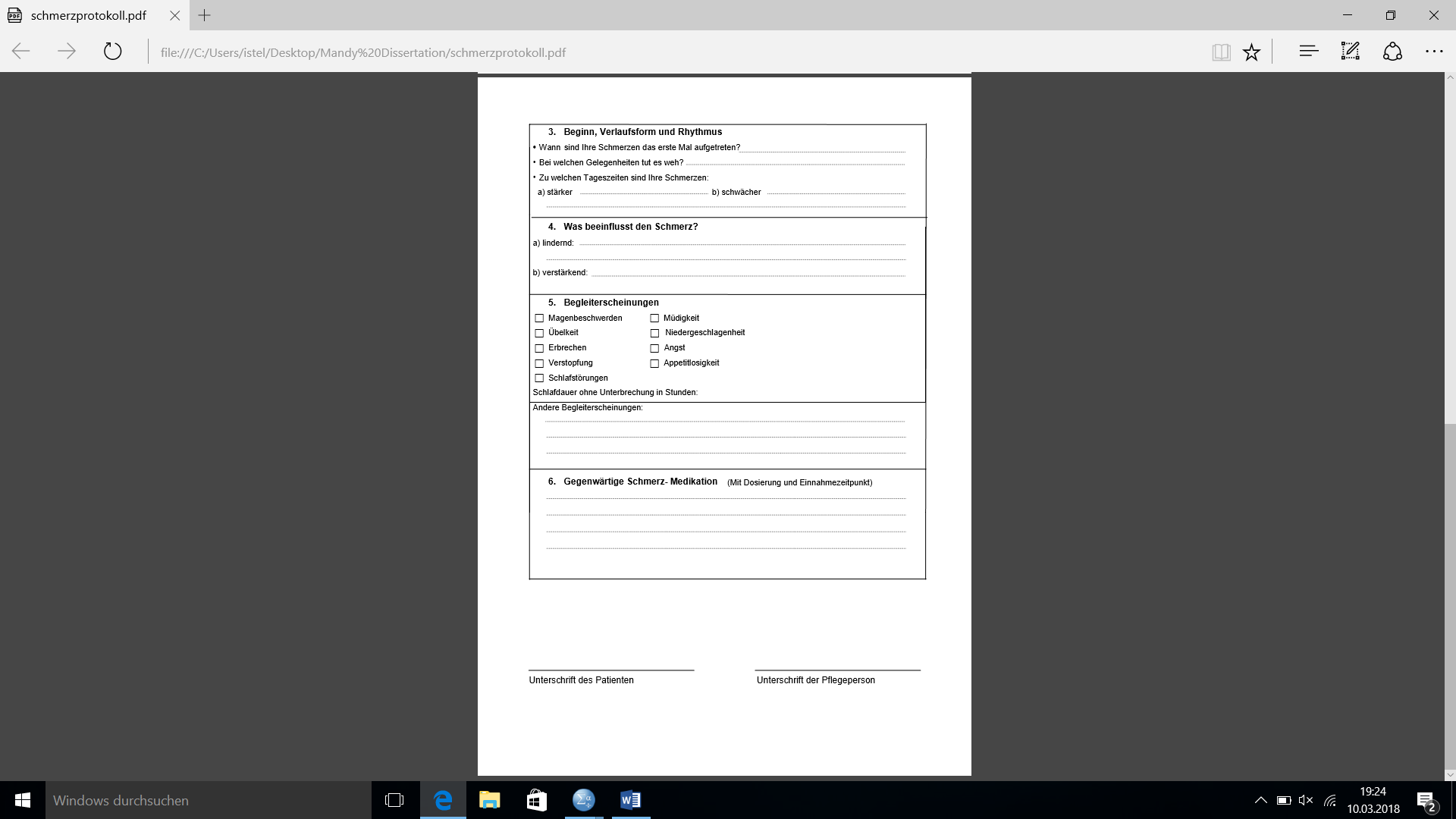


**Appendix 2, Part 1**: Patient information sheet (“Aufklärungsbogen - Studie“)

- **Klinik für Allgemein-, Viszeral- und Gefäßchirurgie** -

Universitätsklinikum - Medizinische Fakultät

OTTO-VON-GUERICKE-UNIVERSITÄT MAGDEBURG

**Stand und Optimierung der schmerztherapeutischen Versorgung am Beispiel der postoperativen Schmerztherapie einer universitären allgemein- und viszeralchirurgischen Station, basierend auf einer etablierten SOP**

**A U F K L Ä R U N G S B O G E N**

- Informationsblatt für Studienteilnehmer -

**Liebe Patientin, lieber Patient !**

Bei Ihnen soll eine chirurgische Behandlung, ggf. eine Operation durchgeführt werden. Dies kann mit Schmerzen verbunden sein. In der Regel sind diese Schmerzen nach 3-5 Tagen behandelt. Allerdings kann es vorkommen, dass im Einzelfall die Schmerzen auch darüber hinaus bestehen bleiben.

Für den Fall wollen wir mit einer im Konsildienst (Arztbesuch anderer Fachrichtung) der Schmerzambulanz dieser Universität langjährig erprobten Verwendung von Schmerzmitteln auch bei Ihnen vorgehen.

Im Rahmen der oben genannten Studie wird unser Pflegepersonal zunächst schon bei Aufnahme einen Schmerzfragebogen Ihnen zur Beantwortung geben.

Alsdann werden wir z.B. nach der OP gemäß Ihren Angaben im Fragebogen wie auch aufgrund der jeweils am Befragungstag aktuell von Ihnen angegebenen Schmerzen gemäß den Möglichkeiten der „standardisierten Vorgehensweise“ („SOP“ = „Standard Operating Procedure“)) Ihre Schmerzen behandeln.

Das Ärzteteam wird entsprechend Ihren Angaben die Medikamente auswählen.

Das Pflegepersonal befragt Sie täglich nach der aktuellen Schmerzstärke auf einer Skala von 0-10. Dabei bedeutet 0= kein Schmerz und 10 bedeutet, dass der Schmerz so stark ist, dass sie „aus dem Fenster springen würden“.

Was müssen Sie dabei tun?

Wir bitten Sie darum, den Ihnen am Aufnahmetag ausgehändigten 2-seitigen Fragebogen auszufüllen.

Außerdem bitten wir Sie, die Frage des Pflegepersonals wie der Ärzte nach den Schmerzen entsprechend Ihrer Wahrnehmung zu beantworten.

Entstehen durch dieses Vorgehen für Sie Nachteile?

Auch ohne diese Studie werden natürlich Ihre Schmerzen behandelt. Wir verbinden mit dieser Untersuchung die Hoffnung, durch ein von allen Mitarbeitern der Station (Ärzte wie Pflegepersonal)

getragenes Vorgehen Ihre Schmerzen in der optimal möglichen Weise zu behandeln.

Worum wir Sie bitten

Eine ordnungsgemäße Überprüfung eines standardisierten Vorgehens ist nicht möglich ohne eine Überprüfung, ob die Schmerztherapie auf der Station auch wirklich gemäß den Vorgaben durchgeführt wurde. Dafür müssen wir Ihre Daten, soweit aus den Patientenakten entnehmbar, gesondert für diese Studie nochmals verwenden dürfen, **um sie in einem Datenregister zu erfassen und auszuwerten**.

Ihre persönlichen Angaben werden selbstverständlich anonym und vertraulich behandelt.

Durch die Untersuchung gewonnene persönliche Daten werden während der Untersuchung verschlüsselt und nach der Auswertung vernichtet.

Die Entscheidung, ob Sie bereit sind, sich in diese Studie einbeziehen zu lassen, ist völlig freiwillig.

Sollten Sie nicht einverstanden sein, ergibt sich daraus für Sie kein Behandlungs- oder Fürsorgenachteil, da dann nach der bisherigen Vorgehensweise Ihre Schmerzen behandelt werden. Eine Schmerztherapie erhalten Sie also- wo notwendig - in jedem Fall.

Ebenso erwächst aus der Studienteilnahme kein Vorteil oder Anspruch für eine finanzielle Vergütung.

Für Fragen stehen wir Ihnen jederzeit gerne zur Verfügung (Tel.: 0391 67 15500) oder Klinikansprechpartner Herr Professor Dr. Meyer (Tel.: 0391 67 15527).

**Appendix 2, Part 2:** Informed consent sheet

(“Einverständniserklärung - Probanden“)

- **Klinik für Allgemein-, Viszeral- und Gefäßchirurgie** -

Universitätsklinikum - Medizinische Fakultät

OTTO-VON-GUERICKE-UNIVERSITÄT MAGDEBURG

**Stand und Optimierung der schmerztherapeutischen Versorgung am Beispiel der postoperativen Schmerztherapie einer universitären allgemein- und viszeralchirurgischen Station, basierend auf einer etablierten SOP**

**Patienteneinwilligungserklärung**

Herr / Frau Dr. ................................. hat mich eingehend über die im Rahmen der oben genannten Studie geplante Datenerfassung und –auswertung zur Verbesserung der mit einer chirurgischen Behandlung in Zusammenhang stehenden Schmerztherapie informiert und alle meine Fragen beantwortet. Das Blatt „Aufklärungsbogen - Informationen für die Studienteilnehmer“ habe ich erhalten, gelesen und verstanden.

Außerdem wurde ich über den Datenschutz meiner persönlichen Daten und der während der Untersuchungen gewonnenen Erkenntnisse aufgeklärt.

Mir ist bekannt, dass ich ohne Angabe von Gründen jederzeit mein Einverständnis zur Datenerfassung und damit Einbeziehung in diese Studie widerrufen kann. Hieraus erwachsen mir keine Nachteile.

Mit der Datenerfassung und –auswertung zur Verbesserung der mit einer chirurgischen Behandlung in Zusammenhang stehenden Schmerztherapie erkläre ich mich hiermit einverstanden.

Studienteilnehmer(in): ...................................................................................................

Name Geburtsdatum

Magdeburg, den ......................................... Unterschrift .....................................

Betreuender Arzt: .............................................................

(Aufklärender Arzt)

Magdeburg, den ......................................... Unterschrift .....................................

**Appendix 3: Algorithm - pain therapy**

Together with the general surgeons (Prof. Meyer) we have agreed on the following procedure as a SOP:

**Part 1: Surgeons and ICU.**

1-3rd postop. Day: fixed scheme:

(a) procedures with less invasiveness (e.g. transurethral surgery):

- PCA pump (45mg piritramide on 22ml NaCl) and additional non-opioid (metamizole max. 5x1g p.o. or as KI i.v.) or butylscopalamine 3x 20mg as KI i.v. (CAVE: glaucoma, increase sympathetic activity).

b) interventions with significant invasiveness (e.g. nephrectomy, cystectomy with ileum conduit, prostatectomy)

ba) in intensive care

- Either with peridural catheter: Continuous ropivacaine 0.2% or 0.375% and additional non-opioid (metamizole, COXII inhibitor).

- without PDK: procedure as in a); additionally morphine perfusor with 5mg metamizole on 48ml NaCl, running rate 2ml/h

- Before transfer to normal ward, initiation of PCA pump with piritramide or morphine if necessary. Continue NSAID regimen "by-the-clock", also continue oral opioid regimens started and reduce successively during course if necessary, co-care by pain service possible.

bb) on normal ward:

- Either with peridural catheter: Continuous ropivacaine 0.2% or 0.375% and additional non-opioid (metamizole, COXII inhibitor).

- Without PdK:

- Antipyretic: metamiczole, COXII inhibitor.

- Piritramide with a PCA pump of 2 mg/ml piritramide for 24 hours.

- From day 2: change PCA pump or piritramide s.c. as needed (7.5 - 15 mg, max 4-hourly).

**Part 2: Consultant and ward physicians.**

After the 3rd postop. day: Pain medication according to pain type!

Modified according to Brinkers *et al*. [7]
